# The impact of β-blockers on outcomes of immune checkpoint inhibitors therapy in advanced lung cancer: a multicenter real-world study

**DOI:** 10.3389/fimmu.2025.1693249

**Published:** 2025-10-21

**Authors:** Lingdan Chang, Haitian Zhang, Yunxia Li, Jilan Yang, Ya Li, Guangming Wang, Jinsong Zhang, Hongjin Shi, Bing Hai

**Affiliations:** ^1^ Department of Respiratory and Critical Care Medicine, The Second Affiliated Hospital of Kunming Medical University, Kunming, Yunnan, China; ^2^ Department of Faculty of Medicine and Health Sciences, UCSI University, Port Dickson, Negeri Sembilan, Malaysia; ^3^ Department of Medical Oncology III, Yunnan Cancer Hospital, Kunming, Yunnan, China; ^4^ Department of Gastrointestinal Oncology, Yunnan Cancer Hospital, Yunnan Cancer Hospital, Kunming, Yunnan, China; ^5^ Department of Respiratory and Critical Care Medicine, Yanan Hospital of Kunming City, Kunming, Yunnan, China; ^6^ School of Information Science & Engineering, Yunnan University, Kunming, China; ^7^ Department of Urology, The Second Affiliated Hospital of Kunming Medical University, Kunming, Yunnan, China

**Keywords:** lung cancer, immunotherapy, immune checkpoint inhibitors, β-adrenergic signaling, β-blockers

## Abstract

**Background:**

Immune checkpoint inhibitors (ICIs) have improved outcomes in advanced lung cancer. β-adrenergic signaling may promote tumor initiation and progression, and β-blockers (BBs) have emerged as anti-tumor sensitizing agents. This study evaluates the impact of BBs use during ICIs treatment in advanced lung cancer.

**Methods:**

This multicenter retrospective real-world study included 462 patients treated with ICIs from June 2019 to December 2024. Patients were divided into BBs and No BBs groups. Primary endpoints were overall survival (OS) and progression-free survival (PFS); efficacy evaluation and objective response rate (ORR) were secondary. Propensity score matching (PSM) balances baseline characteristics. Kaplan–Meier method, Cox, and logistic regression models were used for survival and multivariate analyses. Subgroup analyses assessed clinical factors. A P value < 0.05 is considered statistically significant.

**Results:**

After PSM, 318 patients were included (88 BBs, 230 No BBs). BBs use was associated with longer median PFS (mPFS) (15.8 vs. 11.8 months; HR = 0.67, 95% CI: 0.49–0.92, P = 0.038) and higher ORR (51.1% vs. 35.2%, P = 0.014), but not improved median OS (mOS) (29.0 vs. 31.5 months; HR = 1.38, 95% CI: 0.93–2.03, P = 0.108). BBs use independently predicted improved ORR (OR = 0.45, 95% CI: 0.26–0.78, P = 0.004) and longer PFS (HR = 0.67, 95% CI: 0.49–0.92, P = 0.014). In patients with cardiovascular comorbidities (CVD), BBs use was linked to longer mPFS (15.8 vs. 10.9 months, P = 0.0066) and higher ORR(51.1% vs 27.0%, P<0.001), with no mOS difference (P = 0.82). Among non-small cell lung cancer (NSCLC) patients, mPFS (17.5 vs. 12.3 months, P = 0.04) and ORR (56.0% vs 35.9%, P = 0.004) were also improved in the BBs group, whereas OS did not differ significantly (P = 0.3).

**Conclusion:**

In stage-advanced lung cancer, BBs combined with ICIs were associated with improved ORR and prolonged PFS, but did not significantly improve OS. PFS and ORR benefits were also observed in patients with CVD or NSCLC. Further prospective studies are needed to validate these findings and clarify whether BBs directly contribute to ICIs’ efficacy.

## 1 Introduction

Lung cancer is one of the most common cancers worldwide, with consistently high incidence and mortality rates (1). Early-stage lung cancer often presents without obvious symptoms, and most patients are diagnosed at an advanced stage when clinical manifestations emerge. The overall 5-year survival rate for patients with advanced lung cancer is approximately 20% (2). In recent years, the emergence of immune checkpoint inhibitors (ICIs) has brought about a breakthrough of advanced lung cancer. ICIs function by blocking cytotoxic T-lymphocyte-associated protein 4 (CTLA-4) or the programmed death-1/programmed death-ligand 1 (PD-1/PD-L1) pathways, thereby relieving the inhibition of T cell and natural killer cell activity, restoring their anti-tumor activity, and ultimately suppressing tumor growth and metastasis. ICIs have already been applied in first-line treatment for advanced lung cancer (3, 4).

CD8^+^ T cells are a key component of the anti-tumor immune response and are capable of suppressing cancer cells. When antigens cannot be effectively eliminated, T cells may enter a state of exhaustion (5). T cell exhaustion limits the efficacy of T cell-mediated responses in cancer, resulting in immunotherapy failure or resistance in a substantial proportion of lung cancer patients (6). Preclinical studies have shown that stress-induced β-adrenergic signaling may increase the number and activity of immunosuppressive cells, reduce the production of T cell growth-promoting factors, and inhibit T cell cytotoxicity, ultimately leading to T cell exhaustion (7). Globig et al. found that exhausted T cells expressed higher levels of the adrenergic receptor β1 (ADRB1) in mice with chronic viral infections or cancer. They reported that deletion of ADRB1 in T cells, or pharmacological blockade of ADRB1 using β-blockers (BBs), enhanced the secretion of effector molecules and cytokines and improved T cell function in chronic infection and tumor settings (8). These findings highlight the potential of combining BBs with existing immunotherapies to improve cancer treatment by enhancing cytotoxic T cell function.

Repurposing drugs already approved for other indications is an attractive strategy to optimize treatment outcomes in cancer patients (9). BBs are a class of safe, cost-effective drugs with mild side effects and are widely used for hypertension, cardiovascular diseases, hyperthyroidism, and anxiety (10, 11). Preclinical evidence supports the potential of BBs to enhance lung cancer immunotherapy (8, 12), and there is growing interest in the role of BBs as modulators of tumor initiation, progression, and metastasis (12, 13).

Several clinical studies have found that the use of BBs is associated with prolonged progression-free survival (PFS) in patients with melanoma, while no significant association has been observed with PFS in colorectal or breast cancer (13). In hepatocellular carcinoma, BBs use was also not found to be associated with overall survival (OS), PFS, or objective response rate (ORR) in patients receiving immunotherapy (14). However, a recent large population-based cohort study reported that BBs use was associated with improved cancer-specific survival and recurrence-free interval in triple-negative breast cancer, and reduced risk of distant recurrence (15). Regarding lung cancer, some studies have shown that BBs use is associated with prolonged OS and PFS in non-small cell lung cancer (NSCLC) patients receiving chemoradiotherapy, and may serve as an independent prognostic factor (16–18). In the context of ICIs treatment, the role of BBs in lung cancer remains limited and conflicting. Michael S. Oh et al. reported that BBs use may be associated with improved PFS in NSCLC patients receiving ICIs, but no association with OS (19). While other studies have suggested the use of BBs not associated with OS or PFS, albeit with trends toward improved ORR (11, 20). In contrast, Leshem et al. reported that the use of β1-selective blockers was associated with shorter OS and PFS in NSCLC (21). However, most existing studies are single-center retrospective analyses with limited sample sizes, and their conclusions remain to be further validated.

Given the widespread use of BBs in clinical oncology and their potential immunomodulatory effects, we hypothesized that inhibition of β-adrenergic signaling may enhance the efficacy of immunotherapy in lung cancer. Therefore, we conducted this multicenter real-world retrospective study to investigate the impact of BBs use on the prognosis of advanced lung cancer patients receiving immunotherapy.

## 2 Materials and methods

### 2.1 Study population

This retrospective study included 462 patients with stage III-IV lung cancer who received ICIs treatment at the Second Affiliated Hospital of Kunming Medical University, Yunnan Cancer Hospital, and Yan an Hospital of Kunming city from June 2019 to December 2024. The No BBs group consisted of lung cancer patients who did not use BBs but received at least 2 cycles of ICIs. The BBs group included those who received both BBs and ICIs for at least two cycles. Both groups of patients received other treatments in accordance with clinical guidelines. This study complies with the Helsinki Declaration (22) and has been reviewed by the ethics committees of the three hospitals. The ethics approval numbers are: Shen-PJ-Ke-2024-23, KYLX2024-217, and 2024-232-01, respectively.

### 2.2 Inclusion criteria

(1) Patients diagnosed with lung cancer through pathological and imaging examinations; (2) Patients classified as stage III-IV lung cancer according to the AJCC 8th edition of the TNM staging criteria (23); (3) Patients who received at least two cycles of single-agent or combination ICIs therapy after diagnosis; (4) Age ≥ 18 years, with an Eastern Cooperative Oncology Group (ECOG) performance status score of 0-1 (24) (5); According to the Response Evaluation Criteria in Solid Tumors 1.1 (RECIST 1.1) version (25, 26), there is at least one measurable lesion; (7) No second primary tumors; (8) Complete clinical and follow-up data of the patients are available.

### 2.3 Exclusion criteria

(1) An expected survival of less than 3 months; (2) ECOG score ≥ 2; (3) Use of ICIs and BBs for less than 2 treatment cycles; (4) Presence of immunotherapy contraindications; (5) No measurable lesions; (6) Patients for whom treatment efficacy evaluation is impossible; (7) Patients with incomplete or unavailable data.

### 2.4 Data collection

Clinical data collected included: demographic characteristics (age and gender), body mass index (BMI), smoking index (pack-years), history of CVD (e.g., coronary artery disease and hypertension), and history of diabetes. Collect the tumor-related data, including surgical and radiotherapy history, pathological type, stage, size, metastatic status, baseline treatment situation, pathological and imaging examinations, immunohistochemical features, and ECOG score etc.

### 2.5 Study endpoints

#### 2.5.1 Primary endpoints

OS and PFS. OS was defined as the time from treatment initiation to the date of death from any cause. PFS was defined as the time from treatment initiation to the first documented disease progression or death from any cause, whichever occurred first.

#### 2.5.2 Secondary endpoints

Efficacy evaluation and ORR. According to RECIST 1.1, treatment response was defined as: 1) Complete Response (CR): Disappearance of all target lesions, with all pathological lymph nodes reduced to a short axis of <10 mm, sustained for at least 4 weeks. 2) Partial Response (PR): At least a 30% decrease in the sum of the diameters of target lesions, sustained for at least 4 weeks. 3) Progressive Disease (PD): At least a 20% increase in the sum of the diameters of target lesions, or the appearance of new lesions. 4) Stable Disease (SD): The target lesion neither shows sufficient shrinkage to qualify for PR nor sufficient increase to qualify for PD. ORR: was defined as the proportion of patients achieving CR or PR. In this study, a favorable efficacy evaluation was defined as the CR or PR state. An unfavorable efficacy evaluation was defined as the SD or PD state.

### 2.6 Statistical analyses

Descriptive statistics were performed for categorical and continuous variables. Categorical variables were expressed as numbers (percentages), continuous variables with a normal distribution as mean ± standard deviation, and non-normally distributed variables as median (interquartile range, IQR). Between-group comparisons used the independent-samples t-test or Wilcoxon rank-sum test for continuous variables, and the chi-square or Fisher’s exact test for categorical variables. PSM was conducted with a caliper of 0.2 times the standard deviation of the propensity score, using nearest-neighbor matching at a 1:4 ratio to maximize baseline comparability between groups. PFS and OS were estimated by the Kaplan-Meier method and compared using the log-rank test. Binary logistic regression was used for univariate and multivariate analyses of CR + PR versus PD + SD, and Cox proportional hazards models for PFS and OS to identify independent prognostic factors. Variables with P < 0.1 in univariate analyses and important clinical factors were included in multivariate models. A P value < 0.05 was considered statistically significant. Statistical analyses were performed using SPSS 27.0 and R 4.5.1.

## 3 Results

### 3.1 Baseline characteristics of patients

**Figure 1:**
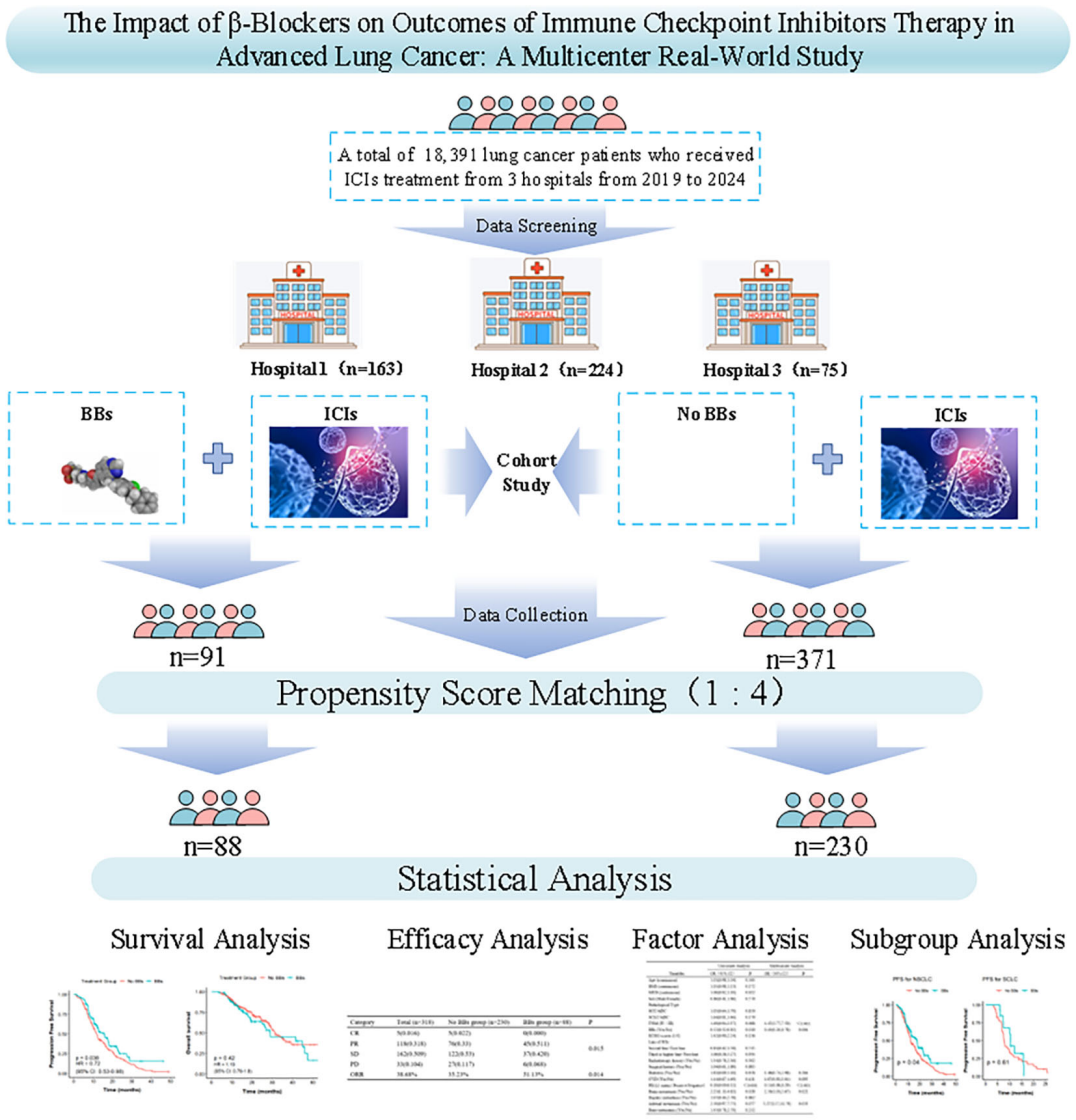
Study design and flow diagram.

A total of 462 lung cancer patients who received ICIs treatment were enrolled in this study, including 163 patients from the Second Affiliated Hospital of Kunming Medical University, 224 from Yunnan Cancer Hospital, and 75 from Yan an Hospital of Kunming city. Based on BBs use, 371 patients were in the No BBs group and 91 in the BBs group. Before PSM, the two groups were comparable in the gender, BMI, smoking index, ECOG score, PDL1 status, TNM stage, immunotherapy line, pathological stage of lung cancer, history of radiotherapy, surgical history, and metastasis sites (all P > 0.05). However, patients in the BBs group were older (65.20 vs 60.82 years, P < 0.001), had a higher proportion of diabetes (P < 0.001), and all had CVD. At the same time, the distribution of treatment lines differed significantly between groups (P = 0.018), with the proportion of first-line treatment higher in the No BBs group (86.79% vs. 78.02%) and the proportion of second-line treatment higher in the BBs group (32.97% vs. 9.43%). To adjust for the above confounding factors, we used 1:4 PSM (covariates included: age, gender, BMI, smoking index, ECOG score, PDL1 status, TNM stage, diabetes, radiotherapy history, surgical history, ICIs treatment lines, maximum diameter of target lesion, metastasis site, etc.) to obtain 230 patients in the No BBs group and 88 in the BBs group. After PSM, baseline characteristics were balanced between groups except for history of CVD (P < 0.001) and diabetes (P = 0.008). **Figure 1** provides the flowchart of the study, and **Table 1** lists the clinical characteristics of the two groups before and after PSM.

**Table 1:** Results of study subjects before and after PSM matching.

Covariates	Before PSM (n=462)			After PSM (n=318)		
	No BBs group (n=371)	BBs group (n=91)	P	No BBs group (n=230)	BBs group (n=88)	P
Sex			0.493			0.964
Female	58 (15.63)	11 (12.09)		31 (13.48)	11 (12.50)	
Male	313 (84.37)	80 (87.91)		199 (86.52)	77 (87.50)	
Pathological Type			0.946			0.991
ADC	145 (39.08)	35 (38.46)		88 (38.26)	34 (38.64)	
SCC	174 (46.90)	42 (46.15)		109 (47.39)	41 (46.59)	
SCLC	52 (14.02)	14 (15.38)		33 (14.35)	13 (14.77)	
ECOG Score			0.723			1.000
0	214(57.68)	50(54.95)		129(56.09)	49(55.68)	
1	157(42.32)	41(45.05)		101(43.91)	39(44.31)	
PDL1 Status			0.842			0.930
Negative	151 (40.70)	36 (39.56)		89 (38.70)	34 (38.64)	
Positive	91 (24.53)	25 (27.47)		61 (26.52)	25 (28.41)	
Unknown	129 (34.77)	30 (32.97)		80 (34.78)	29 (32.95)	
Line of ICIs			0.039			0.522
First line	322 (86.79)	71 (78.00)		196 (85.22)	71 (80.68)	
Second line	35 (9.43)	18 (19.80)		28 (12.17)	15 (17.05)	
Third line/Higher	14 (3.77)	2 (2.20)		6 (2.61)	2(2.27)	
Surgical History			0.691			0.863
No	291 (78.44)	69 (75.82)		179 (77.83)	67 (76.14)	
Yes	80 (21.56)	22 (24.18)		51 (22.17)	21 (23.86)	
Radiotherapy History			0.087			0.344
No	298 (80.32)	65 (71.43)		183 (79.57)	65 (73.86)	
Yes	73 (19.68)	26 (28.57)		47 (20.43)	23 (26.14)	
Diabetes			<0.001			0.008
No	337 (90.84)	64 (70.33)		198 (86.09)	64 (72.73)	
Yes	34 (9.16)	27 (29.67)		32 (13.91)	24 (27.27)	
TNM Stage			0.986			0.707
Stage III	152(40.97)	38(41.76)		87(37.83)	36(40.90)	
Stage IV	219(59.03)	53(58.24)		143(62.17)	52(59.10)	
Brain Metastasis			0.364			0.986
No	322 (86.79)	75 (82.42)		198 (86.10)	75 (85.23)	
Yes	49 (13.21)	16 (17.58)		32 (13.91)	13 (14.77)	
Hepatic Metastasis			0.711			0.875
No	344 (92.72)	86 (94.51)		214 (93.04)	83 (94.32)	
Yes	27 (7.28)	5 (5.49)		16 (6.96)	5 (6.76)	
Adrenal Metastasis			1.000			0.948
No	345 (92.99)	84 (92.31)		214 (93.04)	81 (92.05)	
Yes	26 (7.01)	7 (7.69)		16 (6.96)	7 (7.95)	
Bone Metastasis			0.969			1.000
No	313 (84.37)	76 (83.52)		190 (82.61)	73 (82.95)	
Yes	58 (15.63)	15 (16.48)		40 (17.39)	15 (17.05)	
Age	60.82 ± 8.35	65.20 ± 7.50	<0.001	63.91 ± 7.66)	65.11 ± 7.61	0.210
BMI	23.08 ± 3.39	23.48 ± 3.08	0.298	23.04 ± 3.40)	23.42 ± 3.10	0.368
MTD	5.75 ± 2.68)	5.22 ± 2.34)	0.085	5.36 ± 2.50)	5.27 ± 2.34	0.786
Smoking Index	200.00[0.00, 750.00]	300.00 [0.00, 600.00]	0.762	245.00[0.00, 800.00]	310.00[0.00, 600.00]	0.732
CVD			<0.001			<0.001
No	185 (49.87)	0 (0.0)		97 (42.17)	0 (0.00)	
Yes	186 (50.13)	91 (100.00)		133 (57.83)	88 (100.00)	

Categorical variables were expressed as numbers (percentages), continuous variables with a normal distribution as mean ± standard deviation, and non-normally distributed variables as median (interquartile range). PSM, Propensity score matching; BBs, β-blockers; ADC, Adenocarcinoma; SCC, Squamous cell carcinoma; SCLC, Small cell lung cancer; ECOG, Eastern Cooperative Oncology Group; PDL1, programmed death-ligand 1; ICIs, Immune checkpoint inhibitors; BMI, body mass index; MTD, maximum tumor diameter; CVD, cardiovascular comorbidities.

### 3.2 Efficacy evaluation

The best radiological response was evaluated according to RECIST 1.1. In the No BBs group, the proportions of CR, PD, PR, and SD were 2.2%, 11.7%, 33.0%, and 53.0%, respectively; in the BBs group, the corresponding proportions were 0%, 6.8%, 51.1%, and 42.0%. The distribution of response categories differed significantly between the two groups (P = 0.015). The ORR was 35.23% in the No BBs group and 51.13% in the BBs group, with a statistically significant difference (P = 0.014). These results suggest that BBs use may enhance the therapeutic response to ICIs in lung cancer patients and improve ORR (**Table 2**).

**Table 2:** Treatment efficacy evaluation.

Category	Total (n=318)	No BBs group (n=230)	BBs group (n=88)	P
CR	5(0.016)	5(0.022)	0(0.000)	0.015
PR	118(0.318)	76(0.33)	45(0.511)	
SD	162(0.509)	122(0.53)	37(0.420)	
PD	33(0.104)	27(0.117)	6(0.068)	
ORR	38.68%	35.23%	51.13%	0.014

Data are presented as a number (proportion). CR, Complete Response; PR, Partial Response; PD, Progressive Disease; SD, Stable Disease; ORR, objective response rate; BBs, β-blockers.

To control for confounding factors affecting ORR, univariate and multivariate analyses were performed using a binary logistic regression model comparing patients with CR + PR versus PD + SD (**Supplementary Table S1**). Univariate analysis showed that brain metastasis was significantly associated with poor response (PD+SD) (OR = 2.25, 95% CI: 1.13–4.82, P = 0.028), suggesting it as a negative factor for ORR. Conversely, BBs use (OR = 0.52, 95% CI: 0.32–0.85, P = 0.010) and positive PD-L1 expression (OR = 0.18, 95% CI: 0.09–0.32, P < 0.001) were correlated with higher ORR, indicating protective effects. Multivariate analysis further identified brain metastasis (OR = 2.50, 95% CI: 1.18–5.60, P = 0.022), stage IV TNM (vs. stage III, OR = 4.47, 95% CI: 2.77–7.50, P < 0.001), and adrenal metastasis (OR = 3.27, 95% CI: 1.17–10.76, P = 0.033) as independent risk factors for poor response. Meanwhile, BBs use (OR = 0.45, 95% CI: 0.26–0.78, P = 0.004) and PD-L1 positivity (OR = 0.15, 95% CI: 0.08–0.29, P < 0.001) remained independent protective factors reducing the risk of poor response after adjusting for multiple covariates.

### 3.3 PFS

**Figure 2:**
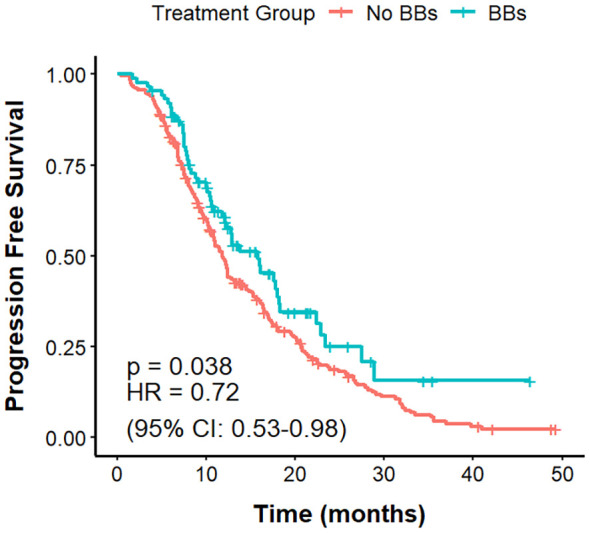
PFS in no BBs vs. BBs groups of lung cancer patients treated with ICIs.

The mPFS was 11.8 months in the No BBs cohort and 15.8 months in the BBs cohort (**Figure 2**). The difference in PFS between the two groups was statistically significant (P = 0.038, HR = 0.72, 95% CI: 0.53–0.98).

Cox proportional hazards models were used to evaluate prognostic factors associated with PFS (**Supplementary Table S2**). In univariate analysis, small cell lung cancer (SCLC) histology (HR = 1.83, 95% CI: 1.26–2.66, P = 0.002), higher ECOG performance status (HR = 1.59, 95% CI: 1.24–2.05, P < 0.001), and brain metastases (HR = 1.61, 95% CI: 1.14–2.27, P = 0.007) were significantly associated with shorter PFS, whereas BBs use (HR = 0.72, 95% CI: 0.53–0.98, P = 0.039) and PD-L1 positivity (HR = 0.41, 95% CI: 0.29–0.56, P < 0.001) were linked to longer PFS. Multivariate analysis, adjusting for variables with P < 0.1 in univariate analysis and key clinical factors, confirmed SCLC histology (HR = 3.08, 95% CI: 2.05–4.62, P < 0.001), ECOG score (HR = 1.66, 95% CI: 1.28–2.15, P < 0.001), brain metastases (HR = 1.45, 95% CI: 1.02–2.08, P = 0.041), adrenal metastases (HR = 1.87, 95% CI: 1.15–3.04, P = 0.012), and bone metastases (HR = 1.51, 95% CI: 1.06–2.15, P = 0.022) as independent predictors of shorter PFS. BBs use (HR = 0.67, 95% CI: 0.49–0.92, P = 0.014) and PD-L1 positivity (HR = 0.32, 95% CI: 0.23–0.46, P < 0.001) remained significant protective factors, with stronger effect sizes after adjustment.

### 3.4 OS

**Figure 3:**
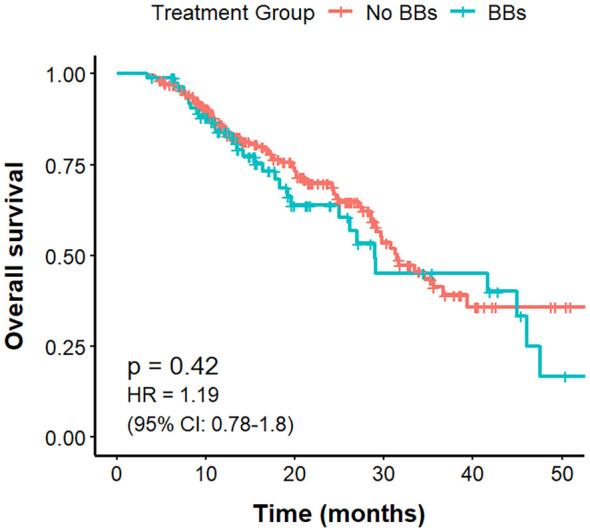
OS in no BBs vs. BBs groups of lung cancer patients treated with ICIs.

The mOS was 31.5 months in the No BBs cohort and 29.0 months in the BBs cohort, with no statistically significant difference between the groups (P = 0.43, HR = 1.19, 95% CI: 0.78–1.79; **Figure 3**).

Univariate and multivariate Cox regression analyses were performed to identify prognostic factors for OS (**Supplementary Table S3**). In the univariate analysis, brain metastases (HR = 1.70, 95% CI: 1.02–2.83, P = 0.040) and bone metastases (HR = 1.63, 95% CI: 1.02–2.60, P = 0.043) were significantly associated with shorter OS, whereas PD-L1 positive expression (HR = 0.53, 95% CI: 0.33–0.86, P = 0.011) was associated with longer OS, suggesting a protective effect. The multivariate model incorporated variables with P < 0.1 from the univariate analysis, and other clinically relevant covariates, revealed SCLC histology (HR = 2.01, 95% CI: 1.08–3.77, P = 0.029) emerged as an independent adverse prognostic factor for OS, while PD-L1 positive expression (HR = 0.74, 95% CI: 0.57–0.95, P = 0.017) remained an independent protective factor. BBs use showed no significant impact on OS (HR = 0.88, 95% CI: 0.65–1.18, P = 0.38).

### 3.5 Subgroup analyses

#### 3.5.1 PFS, OS, and ORR by CVD and BBs status

**Figure 4:**
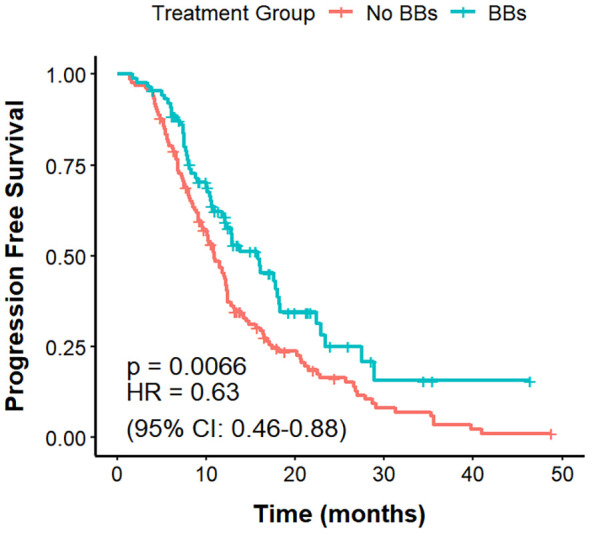
PFS in no BBs vs. BBs groups of lung cancer patients with CVD treated with ICIs.

**Figure 5:**
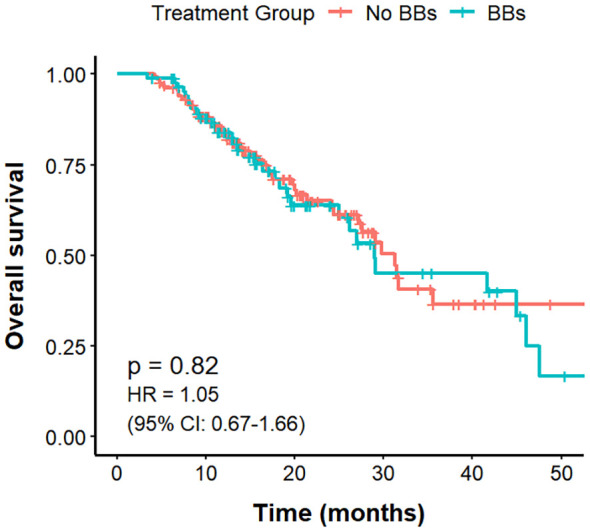
OS in no BBs vs. BBs groups of lung cancer patients with CVD treated with ICIs.

Among the 221 lung cancer patients with CVD, those in the No BBs group (n = 133) had a mPFS of 10.9 months, compared with 15.8 months in the BBs group (n = 88), with a clear difference (P = 0.0066; HR = 0.63, 95% CI: 0.46–0.88). The mOS was 31.2 months in the No BBs group and 29 months in the BBs group, showing no meaningful difference (P = 0.82). The ORR was significantly higher in the BBs group compared with the No BBs group (51.1% vs 27.0%, P<0.001). These findings indicate that, in lung cancer patients with coexisting CVD, treated with BBs exhibit a better short-term efficacy than those without BBs; however, no significant impact on OS was observed (**Figures 4**, **5**, **Table 3**).

**Table 3:** Subgroup analysis of treatment responses according to BBs use.

Subgroup	BBs group	Total	SD+PD	ORR (CR+PR)	P
CVD	No BBs	133	97 (0.729)	36 (0.270)	<0.001
	BBs	88	43 (0.489)	45 (0.511)	
NSCLC	No BBs	195	125 (0.641)	70 (0.359)	0.004
	BBs	75	33 (0.440)	42 (0.560)	
SCLC	No BBs	35	24 (0.686)	11 (0.341)	0.728
	BBs	13	10 (0.769)	3 (0.231)	
Brain Metastasis	No BBs	32	26 (0.813)	6 (0.187)	0.312
	BBs	13	8 (0.615)	5 (0.385)	
Hepatic Metastasis	No BBs	16	10 (0.625)	6 (0.375)	1.000
	BBs	5	3 (0.600)	2 (0.400)	
Adrenal Metastasis	No BBs	16	13 (0.813)	3 (0.187)	0.621
	BBs	7	5 (0.714)	2 (0.286)	
Bone Metastasis	No BBs	40	28 (0.700)	12 (0.300)	0.703
	BBs	15	9 (0.600)	6 (0.400)	

Data are presented as a number (percentage). Comparisons between groups were performed using the χ² test; Fisher’s exact test was applied when expected frequencies were <5. CR, Complete Response; PR, Partial Response; PD, Progressive Disease; SD, Stable Disease; ORR, objective response rate; BBs, β-blockers.

In the No BBs cohort (n=230), patients with CVD (No BBs + CVD, n=133) and those without CVD (No BBs + No CVD, n=97) had mPFS of 10.9 months and 15.3 months, respectively, with a borderline statistical significance (P = 0.051). The mOS was 31.2 months versus 34.6 months, respectively, with no significant difference (P = 0.234). The ORR was significantly higher in the No BBs + No CVD group compared with the No BBs + CVD group (46.4% vs. 27.1%, P = 0.004). These results suggest that some patients with CVD in the no BBs group may be receiving specific treatments that exclude beta-blockers, potentially reducing short-term efficacy (primarily ORR), but have no significant effect on long-term survival (**Supplementary Tables S4**, **S5**, **Supplementary Figures S1**, **S2**).

Further comparison among three groups—BBs + CVD, No BBs + CVD, and No BBs + No CVD — showed that PFS, OS, and ORR were comparable between the BBs + CVD group and the No BBs + No CVD group. These findings indicate that BBs was associated with improved short-term efficacy of ICIs in lung cancer patients with CVD, bringing their outcomes closer to those of patients without CVD (**Supplementary Table** **S4**, **Supplementary Figures S3**, **S4**).

#### 3.5.2 PFS, OS, and ORR in different histologies

**Figure 6:**
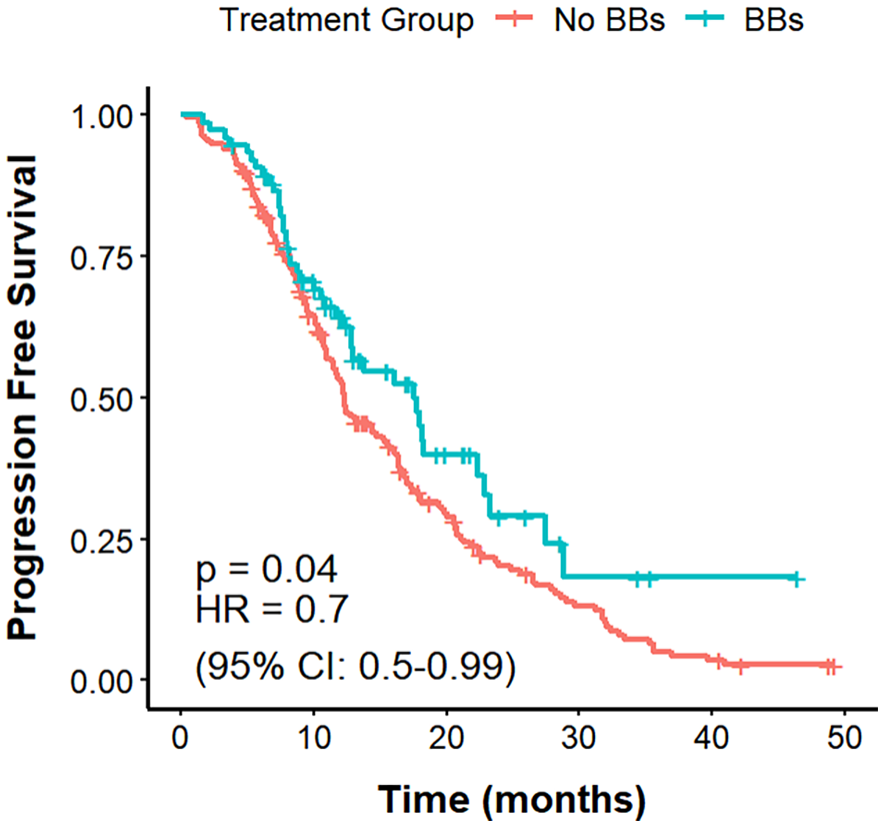
PFS in no BBs vs. BBs groups of NSCLC treated with ICIs.

**Figure 7:**
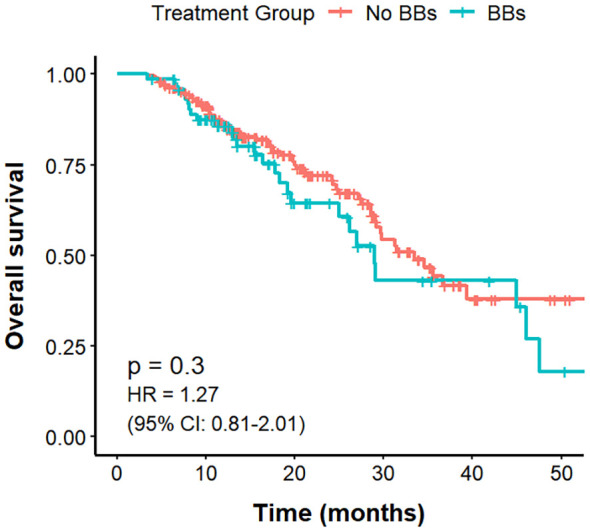
OS in no BBs vs. BBs groups of NSCLC treated with ICIs.

By pathological type, among 270 patients with NSCLC, the BBs group (n = 75) had a longer mPFS compared with the No BBs group (n = 195) (17.5 vs. 12.3 months; P = 0.04, HR = 0.70, 95% CI: 0.40–0.99). However, OS did not differ significantly between the two groups (29.0 vs. 33.5 months; P = 0.30). ORR in the BBs group was notably higher than that in the No BBs group (56.0% vs. 35.9%; P = 0.004). These findings indicate that BBs use in NSCLC is associated with improved PFS but has no evident impact on OS (**Figures 6**, **7**). In 48 patients with SCLC, the mPFS was 7.7 months in the No BBs group (n = 35) and 10.4 months in the BBs group (n = 13), with no statistical difference observed (P = 0.61). The mOS was 30.8 months in the No BBs group and 41.7 months in the BBs group, also without a significant difference (P = 0.56). The ORR likewise showed no difference between the two groups (23.1% vs. 34.1%; P = 0.728). These results suggest a potential clinical benefit of BBs in SCLC, though confirmation in larger cohorts is warranted due to the limited sample size (**Supplementary Figures S5**, **S6**, **Table 3**).

#### 3.5.3 PFS, OS, and ORR by distant metastasis sites

In subgroup analyses by metastatic site, BBs use did not demonstrate a clear survival and efficacy advantage. Among 45 patients with brain metastases, the BBs group (n = 13) had an mPFS of 8.8 months versus 9.4 months in the No BBs group (n = 32) (P = 0.43); mOS was 19.5 vs. 29.2 months, respectively (P = 0.43). In 21 patients with liver metastases, the BBs group (n = 5) was too small to reach mPFS or mOS, whereas the No BBs group (n = 16) had an mPFS of 9.1 months (P = 0.48) and an mOS of 29.8 months (P = 0.39). Among 23 patients with adrenal metastases, the mPFS was 10.4 months in the BBs group (n = 7) and 8.4 months in the No BBs group (n = 16) (P = 0.46). The No BBs group had an mOS of 39.4 months, while mOS was not reached in the BBs group due to insufficient events (P = 0.64). In 55 patients with bone metastases, the BBs group (n = 15) had an mPFS of 15.8 months compared with 8.8 months in the No BBs group (n = 40) (P = 0.35); mOS was 29 months in both groups (P = 0.99). The ORR did not differ significantly between the BBs and No BBs groups in any of these metastatic subgroups (all P > 0.05). Given the small sample sizes in these subgroups, these findings should be interpreted with caution (**Supplementary Figure S7**, **Table 3**).

## 4 Discussion

This study investigated the association between BBs and the clinical outcomes of ICIs in patients with advanced lung cancer. We found that in patients treated with ICIs, BBs use was linked to longer PFS and higher ORR, but showed no effect on OS. This finding is consistent with the results reported by Michael S. Oh et al., who suggested that BBs could extend PFS (19), and aligns with reports in other cancers, such as melanoma, renal cell carcinoma, urothelial carcinoma, and breast cancer, where BBs have been suggested to enhance ICIs efficacy (12, 13, 27), suggesting a potential immune-sensitizing effect of BBs. Unlike most previous single-center, small-sample retrospective studies, our study was based on multicenter real-world data with a larger sample size, and applied PSM and multivariable regression analyses to minimize confounding, providing more robust evidence. Duarte Mendes et al. also observed a favorable trend of BBs use in ICIs-treated lung cancer patients, but it was not statistically significant (11); our larger sample and rigorous methodology likely yield more reliable results.

β-adrenergic receptors (β-AR) are widely expressed in tumor cells, immune cells, and vascular endothelial cells. They promote an immunosuppressive microenvironment by activating multiple pathways, including cAMP/PKA, MAPK, and PI3K/AKT, and can induce exhaustion of CD8+ T cells (28–31). For example, β-AR activation increases intracellular cAMP levels, which activate protein kinase A (PKA). PKA phosphorylates key signaling molecules, disrupting T cell receptor-mediated signaling and inhibiting CD8+ T cell activation, proliferation, glucose uptake, and glycolysis (32). BBs can reverse stress-induced immunosuppression by blocking β-AR signaling, restoring CD8+ T cell antitumor activity (33). In animal models, combining propranolol with anti-PD-1 therapy enhances tumor control (34). Our findings in lung cancer patients provide strong support for an independent association between BBs use and improved ORR and PFS.

Our study is a multicenter retrospective analysis of 462 patients with advanced lung cancer from three hospitals. In the unmatched original cohort, the BBs group exhibited more unfavorable baseline characteristics, including older age, a higher prevalence of diabetes, and universal presence of underlying CVD, indicating a generally poorer health status and greater comorbidity burden. Furthermore, the BBs group had a lower proportion of patients receiving ICIs as first-line therapy, while previous studies have shown ICIs to be more effective in the first-line setting (35, 36). These factors may have introduced bias favoring the No BBs group. Therefore, to control for these potential confounding factors, PSM was performed, resulting in 230 patients in the No BBs group and 88 patients in the BBs group. After matching, baseline characteristics were comparable between the two groups except for differences in history of CVD (P < 0.001) and diabetes (P = 0.008). Notably, the imbalance in CVD stems from the clinical indications for BBs, which are primarily prescribed for hypertension, arrhythmia, heart failure, and other cardiovascular conditions (10). Thus, this imbalance reflects clinical practice realities rather than a methodological flaw. However, in subsequent multivariate Cox and logistic regression analyses, diabetes and CVD were not identified as independent prognostic factors for PFS, OS, or ORR, suggesting a limited impact of these differences on the primary outcomes.

Using logistic regression and Cox proportional hazards models, we identified independent risk and protective factors for ORR, PFS, and OS in lung cancer patients receiving ICIs therapy, which may provide valuable guidance for treatment and prognosis. Regarding efficacy, the BBs group showed a higher ORR than the No BBs group (51.13% vs. 35.23%, P = 0.014), indicating a reduced risk of disease stabilization or progression. Multivariate logistic regression analysis confirmed BBs use as an independent protective factor for ORR (OR = 0.45, 95% CI: 0.26–0.78, P = 0.004). Similarly, the Cox proportional hazards model indicated a protective effect of BBs on PFS (HR = 0.67, 95% CI: 0.49–0.92, P = 0.014), but the association with OS did not reach statistical significance (HR = 0.82, 95% CI: 0.56–1.21, P = 0.32). These results suggest that BBs may enhance early treatment response by modulating sympathetic nervous system activity and improving the immune microenvironment, while their long-term survival benefit may be attenuated by other confounding factors (37). PD-L1 positivity emerged an independent protective factor for ORR, PFS, and OS, consistent with the current consensus in immunotherapy research (38, 39), underscoring its reliability and clinical value as a biomarker and the robustness of our data. In addition, our study found that an elevated ECOG score was an independent risk factor for PFS, with higher scores associated with reduced benefit. ECOG is a recognized prognostic factor for PFS across cancers, patients with poor ECOG scores often receive less intensive treatment, which may affect cancer control and survival. Those in poorer overall health also tend to have more comorbidities and healthcare-related issues; thus, the observed close association between ECOG status and PFS is not surprising (40–42). SCLC histology was also confirmed as an independent risk factor for both PFS and OS, consistent with its aggressive biology and poor prognosis. Literature reports a 5-year survival rate below 7% in SCLC, and its rapid progression, early metastasis, and relatively low response rate to immunotherapy make it one of the lung cancer subtypes with the poorest prognosis (43, 44). Meanwhile, the presence of distant metastases further highlights the complexity of disease progression: brain metastasis was identified as an independent risk factor for both ORR and PFS, while adrenal metastasis and TNM stage IV were independent risk factors for ORR. These findings suggest that distant metastases not only indicate extensive disease spread and aggressive tumor biology but may also be linked to high tumor burden, a strongly immunosuppressive microenvironment, or limited drug penetration (45–47).

This study further explored the prognostic differences of BBs use among patients with varying clinical characteristics through subgroup analysis. Among lung cancer patients with comorbid CVD, BBs use was associated with prolonged mPFS (15.8 vs. 10.9 months; P = 0.0066) and an increased ORR (51.1% vs. 27.0%; P <0.001) relative to the No BBs group, while no difference was observed in OS. Within the No BBs group, patients with CVD had slightly lower ORR than those without CVD, but PFS and OS were comparable. Comparison between the BBs group and the No BBs + No CVD group revealed no significant differences in PFS, OS, or ORR. These findings suggest that some patients with CVD in the no BBs group may be receiving specific treatments that exclude beta-blockers, potentially reducing short-term efficacy. Meanwhile, BBs may influence tumor progression and mitigate CVD complications (17). Relevant preclinical studies indicate that chronic stress associated with CVD leads to sustained activation of adrenergic signaling, which reduces immune cell infiltration in the tumor microenvironment and suppresses antitumor immunity and tumor cell apoptosis (48, 49); and BBs can antagonize these processes. BBs may partly contribute to improved short-term efficacy in patients with CVD, but further studies are needed to confirm whether this reflects a true synergistic effect with ICIs or reduced efficacy in CVD patients not receiving BBs or other confounding variables.

Subgroup analysis by lung cancer histology showed that in NSCLC patients, those using BBs had a longer mPFS (17.5 vs. 12.3 months, P = 0.04) and a higher ORR (56.0% vs. 35.9%; P = 0.004) than the No BBs group, further supporting the potential role of BBs as ICIs sensitizers. In SCLC patients, BBs use showed trends toward prolonged PFS and OS (PFS: 10.4 vs. 7.7 months; OS: 41.7 vs. 30.8 months), but these differences were not statistically significant (P > 0.05). Given the small sample size in SCLC (n = 48) and limited statistical power, these findings warrant cautious interpretation. SCLC is a highly aggressive tumor characterized by high tumor mutational burden, but with low immune cell infiltration, reduced major histocompatibility complex expression, presence of immunosuppressive cells and cytokines, ischemic tumor regions, and rapid growth enabling immune evasion, all of which limit ICI efficacy (50–52). Real-world studies show that durable benefit from ICIs occurs only in a minority of SCLC patients (53). Our results provide preliminary evidence for further exploration of BBs in SCLC immunotherapy, warranting validation in larger cohorts. Additionally, subgroup analyses by sites of distant metastasis revealed no clinically meaningful differences, possibly due to limited sample sizes.

This study has several limitations. First, although PSM was applied, significant differences in the CVD and diabetes history remained between groups. More stringent matching would have led to substantial sample loss and reduced statistical power. To address residual confounding, multivariate Logistic and Cox regression analyses were performed for further adjustment. Second, as a retrospective study, unmeasured confounders such as BBs type, dosage, adherence, psychological stress, and inflammatory markers may still exist despite PSM and multivariable adjustments. Prospective cohort studies and preclinical experiments are needed to confirm these findings. Third, due to a lack of data on lung cancer patients using BBs for non-CVD reasons, subgroup analyses in patients without CVD were not conducted; future studies should include this population. Finally, the relatively small sample size in the BBs group, particularly in subgroup analyses, limits statistical power and calls for larger studies to validate these results. Despite its limitations, the study based on multicenter real-world, provides a foundation for future prospective studies and supports further exploration of BBs as immune-modulating agents in clinical practice.

## 5 Conclusion

This multicenter real-world retrospective study found that among patients with stage III–IV lung cancer treated with ICIs, the use of BBs exhibited a higher ORR and longer PFS, but no improvement in OS. Subgroup analysis showed that BBs also improved PFS and ORR in patients with CVD or NSCLC. Multivariate analysis confirmed BBs as an independent protective factor for ORR and PFS. However, it is still uncertain whether these benefits are directly attributable to BBs or may be influenced by other factors, including concomitant CVD treatments and other confounding factors. Further prospective studies and clinical trials are needed to clarify the causal relationship and validate these results.

## Data Availability

The original contributions presented in the study are included in the article/**Supplementary Material**. Further inquiries can be directed to the corresponding authors.
