# Supplementary material for: The impact of β-blockers on outcomes of immune checkpoint inhibitors therapy in advanced lung cancer: a multicenter real-world study
Source: Front Immunol. 2025 Oct 21;16:1693249. doi: 10.3389/fimmu.2025.1693249 (PMC12583203; doi:10.3389/fimmu.2025.1693249)
Supplement: Supplementary file 1 [file Supplementaryfile1.doc]

Table S1: Univariate and Multivariate Analysis of Prognostic Factors Associated with ORR

| Variables | Univariate Analysis | | Multivariate Analysis | |
| --- | --- | --- | --- | --- |
|  | OR（95% CI） | P | OR（95% CI） | P |
| Age (continuous) | 1.01(0.98,1.04) | 0.564 |  |  |
| BMI (continuous) | 1.05(0.98,1.13) | 0.172 |  |  |
| MTD (continuous) | 1.00(0.92,1.10) | 0.922 |  |  |
| Sex (Male/Female) | 0.86(0.41,1.60) | 0.579 |  |  |
| Pathological Type |  |  |  |  |
| SCC/ADC | 1.05(0.64,1.70) | 0.850 |  |  |
| SCLC/ADC | 1.64(0.81,3.46) | 0.179 |  |  |
| TNM (IV / III) | 1.49(0.94,2.37) | 0.088 | 4.47(2.77,7.50) | ＜0.001 |
| BBs (Yes/No) | 0.52(0.32,0.85) | 0.010 | 0.45(0.26,0.78) | 0.004 |
| ECOG scores (1/0) | 1.42(0.90,2.24) | 0.136 |  |  |
| Line of ICIs |  |  |  |  |
| Second line/ First line | 0.81(0.42,1.56) | 0.515 |  |  |
| Third or higher line/ First line | 1.06(0.26,5.27) | 0.934 |  |  |
| Radiotherapy history (Yes/No) | 1.34(0.78,2.36) | 0.302 |  |  |
| Surgical history (Yes/No) | 1.04(0.61,1.80) | 0.885 |  |  |
| Diabetes (Yes/No) | 1.63(0.89,3.10) | 0.658 | 1.46(0.74,2.98) | 0.284 |
| CVD (Yes/No) | 1.14(0.87,1.49) | 0.121 | 1.67(0.92,3.04) | 0.095 |
| PD-L1 status（Positive/Negative） | 0.18(0.09,0.32) | ＜0.001 | 0.15(0.08,0.29) | ＜0.001 |
| Brain metastasis (Yes/No) | 2.25(1.13,4.82) | 0.028 | 2.50(1.18,5.67) | 0.022 |
| Hepatic metastasis (Yes/No) | 1.07(0.44,2.78) | 0.882 |  |  |
| Adrenal metastasis (Yes/No) | 2.50(0.97,7.75) | 0.077 | 3.27(1.17,10.76) | 0.033 |
| Bone metastasis (Yes/No) | 1.43(0.78,2.70) | 0.252 |  |  |

ORR: objective response rate. BMI: body mass index. MTD: maximum tumor diameter. ADC: Adenocarcinoma. SCC: Squamous cell carcinoma. SCLC: Small cell lung cancer. BBs: β-blockers. ECOG: Eastern Cooperative Oncology Group. CVD: cardiovascular comorbidities. PDL1: programmed death-ligand 1. ICIs: Immune checkpoint inhibitors.

Table S2: Univariate and Multivariate Analysis of Prognostic Factors Associated with PFS.

| Variables | Univariate Analysis | | Multivariate Analysis | |
| --- | --- | --- | --- | --- |
|  | HR（95% CI） | P | HR（95% CI） | P |
| Age (continuous) | 1.00(0.98,1.02) | 0.940 |  |  |
| BMI (continuous) | 1.01(0.98,1.05) | 0.474 |  |  |
| MTD (continuous) | 1.00(0.95,1.05) | 0.991 |  |  |
| Sex (Male/Female) | 1.01(0.70,1.45) | 0.972 |  |  |
| Pathological Type |  |  |  |  |
| SCC/ADC | 0.98(0.75,1.29) | 0.908 | 1.25(0.93,1.68) | 0.147 |
| SCLC/ADC | 1.83(1.26,2.66) | 0.002 | 3.08(2.05,4.62) | < 0.001 |
| TNM (IV / III) | 1.16(0.89,1.49) | 0.269 |  |  |
| BBs (Yes/No) | 0.72(0.53,0.98) | 0.039 | 0.67(0.49,0.92) | 0.014 |
| ECOG scores (1/0) | 1.59(1.24,2.05) | ＜0.001 | 1.66(1.28,2.15) | < 0.001 |
| Line of ICIs |  |  |  |  |
| Second line/ First line | 1.10(0.77,1.57) | 0.599 |  |  |
| Third or higher line/ First line | 1.60(0.71,3.61) | 0.260 |  |  |
| Radiotherapy history (Yes/No) | 1.27(0.94,1.72) | 0.124 |  |  |
| Surgical history (Yes/No) | 0.98(0.73,1.32) | 0.875 |  |  |
| Diabetes (Yes/No) | 1.08(0.77,1.50) | 0.658 | 0.99(0.70, 1.41) | 0.963 |
| CVD (Yes/No) | 1.14(0.87,1.49) | 0.345 | 0.86(0.61, 1.21) | 0.388 |
| PD-L1 status（Positive/Negative） | 0.41(0.29,0.56) | ＜0.001 | 0.32(0.23,0.46) | ＜0.001 |
| Brain metastasis (Yes/No) | 1.61(1.14,2.27) | 0.007 | 1.45(1.02,2.08) | 0.041 |
| Hepatic metastasis (Yes/No) | 1.03(0.61,1.74) | 0.917 |  |  |
| Adrenal metastasis (Yes/No) | 1.60(0.97,2.58) | 0.057 | 1.87(1.15,3.04) | 0.012 |
| Bone metastasis (Yes/No) | 1.37(0.97,1.89) | 0.061 | 1.51(1.06,2.15) | 0.022 |

PFS: progression-free survival. BMI: body mass index. MTD: maximum tumor diameter. ADC: Adenocarcinoma. SCC: Squamous cell carcinoma. SCLC: Small cell lung cancer. BBs: β-blockers. ECOG: Eastern Cooperative Oncology Group. CVD: cardiovascular comorbidities. PDL1: programmed death-ligand 1. ICIs: Immune checkpoint inhibitors.

Table S3: Univariate and Multivariate Analysis of Prognostic Factors Associated with OS.

| Variables | Univariate Analysis | | Multivariate Analysis | |
| --- | --- | --- | --- | --- |
|  | HR（95% CI） | P | HR（95% CI） | P |
| Age (continuous) | 1.00(0.97,1.02) | 0.940 |  |  |
| BMI (continuous) | 0.98(0.93,1.04) | 0.526 |  |  |
| MTD (continuous) | 1.01(0.94,1.08) | 0.991 |  |  |
| Sex (Male/Female) | 1.01(0.70,1.45) | 0.850 |  |  |
| Pathological Type |  |  |  |  |
| SCC/ADC | 0.97(0.65,1.47) | 0.876 | 1.27(0.80,2.01) | 0.312 |
| SCLC/ADC | 1.67(0.94,2.95) | 0.080 | 2.01(1.08,3.77) | 0.029 |
| TNM (IV / III) | 1.36(0.92,2.03) | 0.127 |  |  |
| BBs (Yes/No) | 1.18(0.78,1.79) | 0.435 |  |  |
| ECOG scores (1/0) | 1.4(0.95,2.06) | 0.087 | 0.99(0.67,1.47) | 0.964 |
| Line of ICIs |  |  |  |  |
| Second line/ First line | 1.29(0.78,2.15) | 0.326 |  |  |
| Third or higher line/ First line | 0.60(0.08,3.61) | 0.607 |  |  |
| Radiotherapy history (Yes/No) | 1.14(0.709,1.82) | 0.595 |  |  |
| Surgical history (Yes/No) | 1.18(0.77,1.82) | 0.451 |  |  |
| Diabetes (Yes/No) | 1.35(0.85,2.16) | 0.207 | 1.03(0.62, 1.71) | 0.915 |
| CVD (Yes/No) | 1.37(0.90,2.1) | 0.142 | 1.26(0.80, 1.99) | 0.311 |
| PD-L1 status（Positive/Negative） | 0.53(0.33,0.86) | 0.011 | 0.74(0.57, 0.95) | 0.017 |
| Brain metastasis (Yes/No) | 1.70(1.02,2.83) | 0.040 | 1.56(0.92, 2.65) | 0.096 |
| Hepatic metastasis (Yes/No) | 1.45(0.73,2.89) | 0.284 |  |  |
| Adrenal metastasis (Yes/No) | 1.60(0.78,3.29) | 0.205 |  |  |
| Bone metastasis (Yes/No) | 1.63(1.02,2.60) | 0.043 | 1.46(0.87,2.45) | 0.155 |

OS: overall survival. BMI: body mass index. MTD: maximum tumor diameter. ADC: Adenocarcinoma. SCC: Squamous cell carcinoma. SCLC: Small cell lung cancer. BBs: β-blockers. ECOG: Eastern Cooperative Oncology Group. CVD: cardiovascular comorbidities. PDL1: programmed death-ligand 1. ICIs: Immune checkpoint inhibitors.

Table S4: Subgroup Analysis of Treatment Efficacy Evaluation (Three-Group Comparison).

| Category | BBs + CVD (n=88) | No BBs + CVD (n=133) | No BBs + No CVD (n=97) | P |
| --- | --- | --- | --- | --- |
| CR | 0 (0.000) | 2(0.015) | 3(0.031) | 0.002 |
| PR | 45 (0.511) | 34(0.256) | 42(0.433) |
| SD | 37 (0.420) | 80(0.601) | 42(0.433) |
| PD | 6 (0.068) | 17(0.128) | 10(0.103) |
| ORR (CR+PR) | 51.13% | 27.07% | 46.39% | ＜0.001 |

Data are presented as a number (percentage). Comparisons between groups were performed using the χ² test; Fisher’s exact test was applied when expected frequencies were <5. CR: Complete Response. PR: Partial Response. PD: Progressive Disease. SD: Stable Disease. ORR: objective response rate. BBs: β-blockers.

Table S5: Subgroup Analysis of ORR by CVD and BBs status (Pairwise Comparisons).

| Comparison | Subgroup | Total (n) | SD+PD | ORR (CR+PR) | P |
| --- | --- | --- | --- | --- | --- |
| 1 | BBs + CVD | 88 | 43(0.489) | 45(0.511) | ＜0.001 |
|  | No BBs + CVD | 133 | 97 (0.729) | 36 (0.271) |  |
| 2 | No BBs + CVD | 133 | 97 (0.729) | 36 (0.271) | 0.004 |
|  | No BBs + No CVD | 97 | 52 (0.536) | 45 (0.464) |  |
| 3 | BBs + CVD | 88 | 43(0.489) | 45(0.511) | 0.619 |
|  | No BBs + No CVD | 97 | 52 (0.536) | 45 (0.464) |  |

Data are presented as a number (percentage). Comparisons between groups were performed using the χ² test. CR: Complete Response. PR: Partial Response. PD: Progressive Disease. SD: Stable Disease. ORR: objective response rate. BBs: β-blockers.


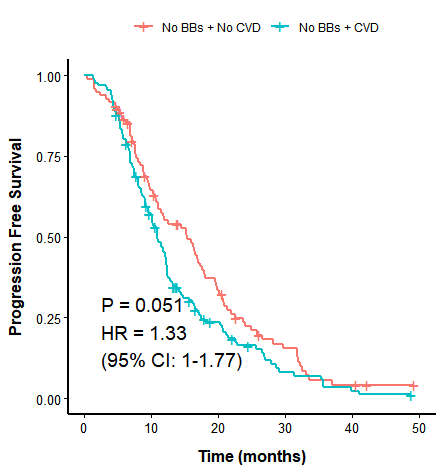


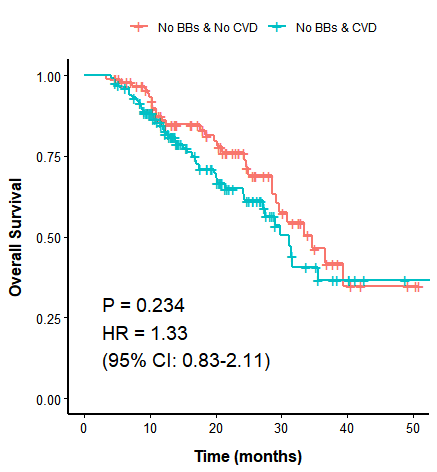


Figure S1 PFS in the No BBs Group Stratified by CVD Status.

Figure S2 OS in the No BBs Group Stratified by CVD Status.


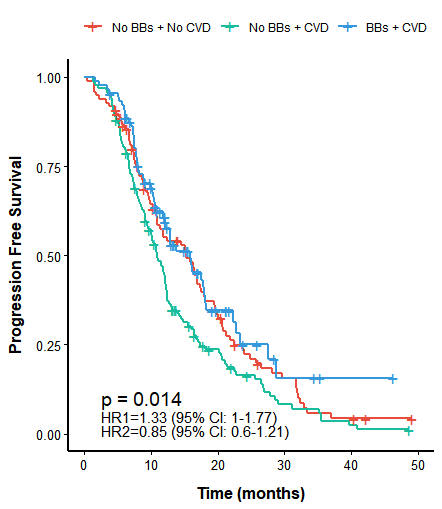


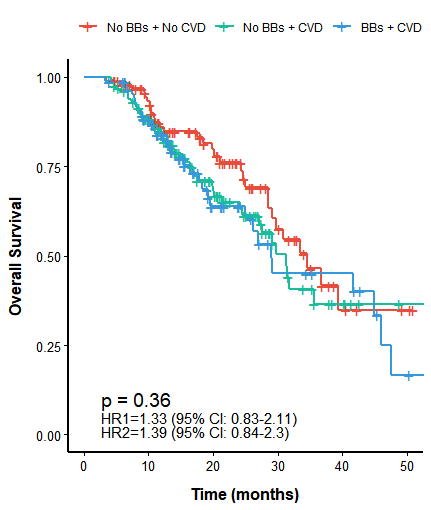


Figure S3 PFS by CVD and BBs status.

HR1: No BBs + CVD vs No BBs + No CVD.

HR2: BBs + CVD vs No BBs + No CVD.

Figure S4 OS by CVD and BBs status.

HR1: No BBs + CVD vs No BBs + No CVD.

HR2: BBs + CVD vs No BBs + No CVD.


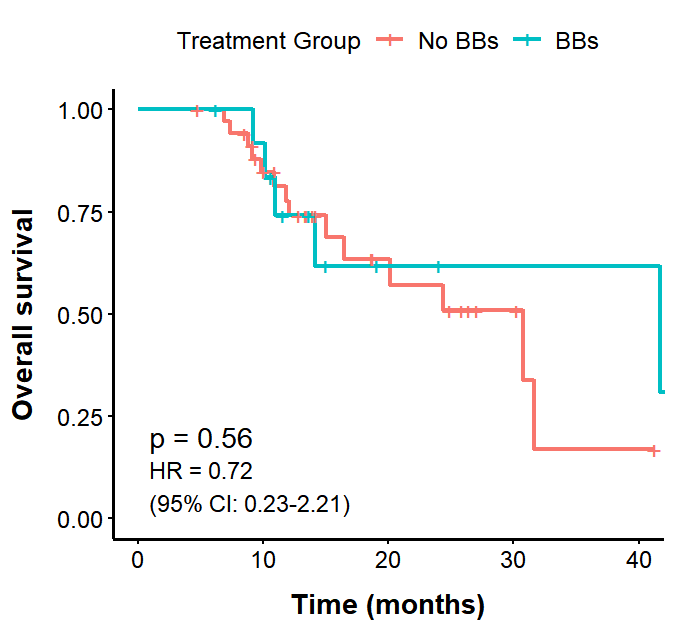


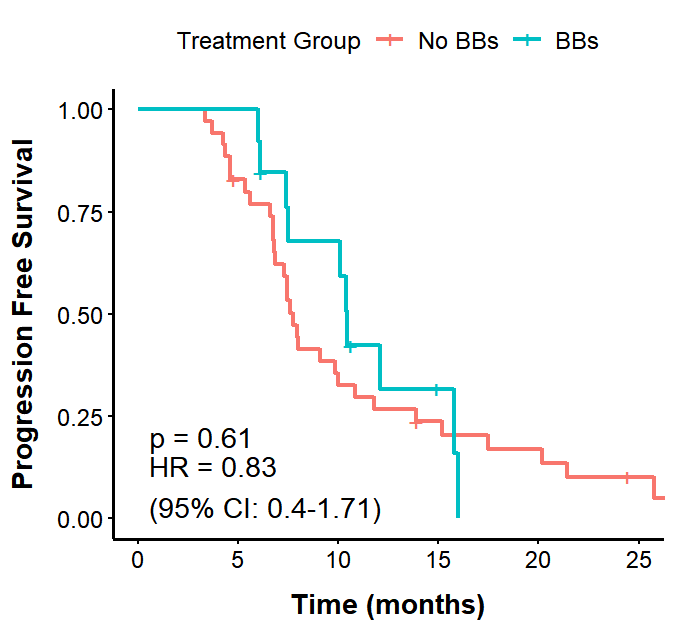


Figure S6 OS in No BBs vs. BBs Groups of SCLC Treated with ICIs.

Figure S5 PFS in No BBs vs. BBs Groups of SCLC Treated with ICIs.


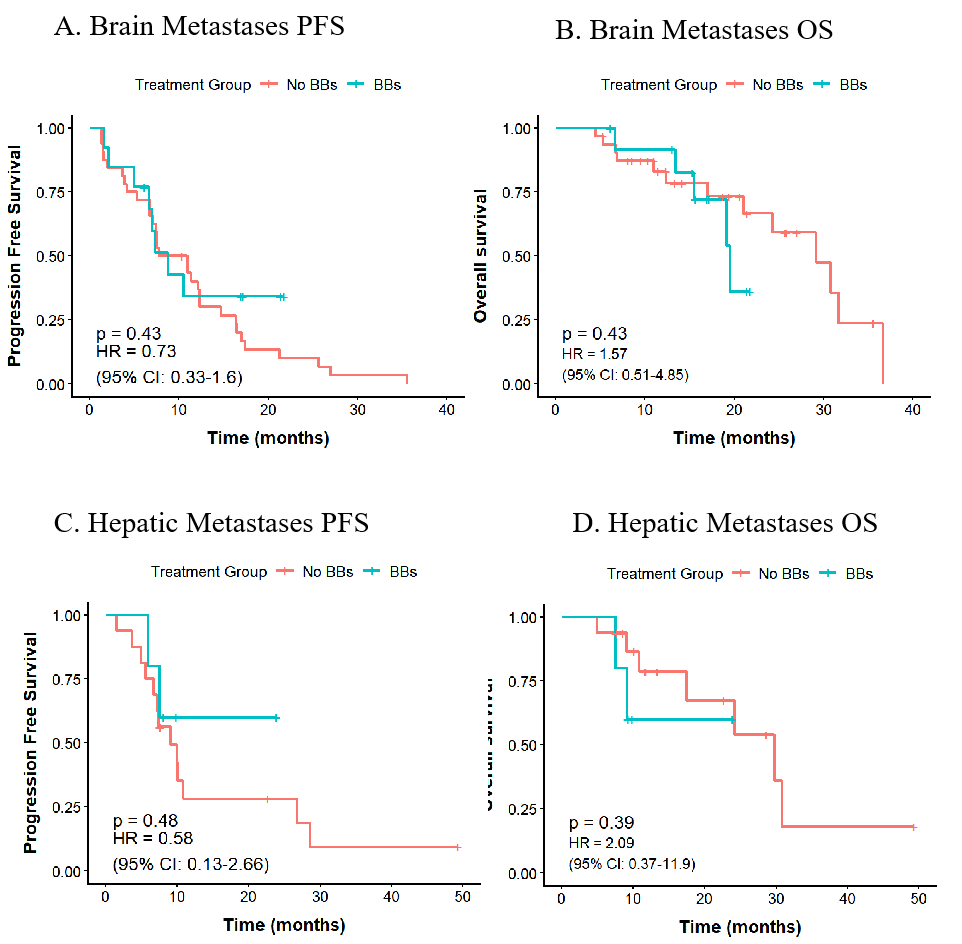


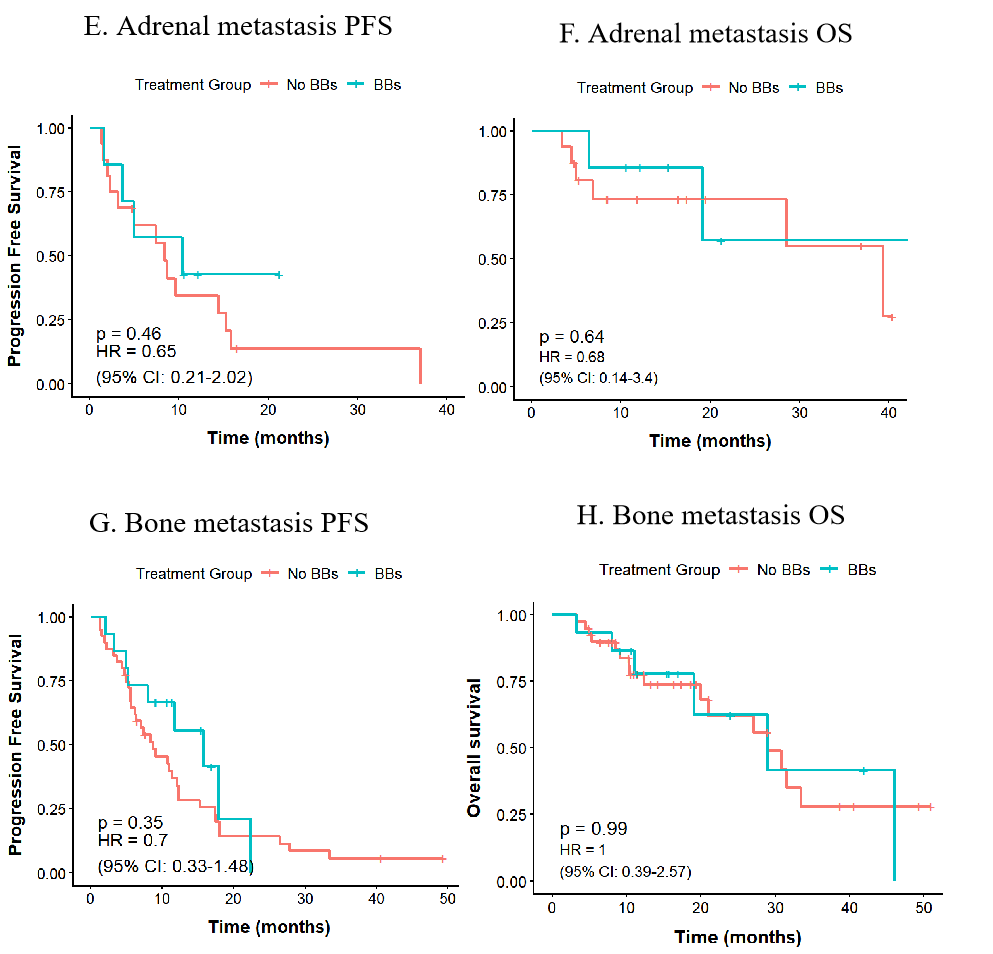


Figure S7 PFS and OS in No BBs vs. BBs Groups of Different Distant Metastasis Sites Treated with ICIs.
